# Cutaneous manifestations of dialysis-associated steal syndrome in a patient with end-stage renal disease

**DOI:** 10.1016/j.jdcr.2024.09.017

**Published:** 2024-10-09

**Authors:** Kristina Blegen, Maryam Niazi, Corley Pruneda, Michelle Tarbox

**Affiliations:** a Department of Dermatology, Texas Tech University Health Sciences Center, Lubbock, Texas; b School of Medicine, Texas Tech University Health Sciences Center, Lubbock, Texas

**Keywords:** dialysis, dystrophy, perforating, renal, vascular

## Introduction

Dialysis-associated steal syndrome (DASS) is a feared complication of arteriovenous fistula (AVF) placement for hemodialysis characterized by diversion of arterial blood flow and hypoperfusion of the distal extremity.^1^ Physiologic or asymptomatic steal occurs in up to 90% of cases of dialysis access, whereas clinically significant DASS requiring surgical intervention is seen in only 1% to 8% of patients and may present with cyanosis, erythema, ischemic ulcers, distal limb pain, paresthesias, weakness, and gangrene in more severe cases.^1^^,^^2^ Herein we discuss a patient with end-stage renal disease (ESRD) whose cutaneous findings and severe digital pain lead to the diagnosis of DASS.

## Case report

A 47-year-old right-hand dominant immunosuppressed woman with a kidney transplant because of ESRD from diabetes mellitus, previous failed AVF placement in the left side of the upper extremity, and current right side of the upper-extremity AVF, presented to the dermatology clinic with a painful 6 mm papule with a central keratotic plug on the right dorsal fourth digit and a dystrophic fourth fingernail. The skin of her dorsal aspect of the right hand and wrist was slightly hypopigmented. The patient was no longer receiving hemodialysis after her successful renal transplant. A shave biopsy of the digital skin lesion confirmed the diagnosis of acquired perforating dermatosis, or Kyrle disease. She had no perforating skin lesions elsewhere at presentation. Fungal culture of the nail clipping was negative.

**Fig 1:**
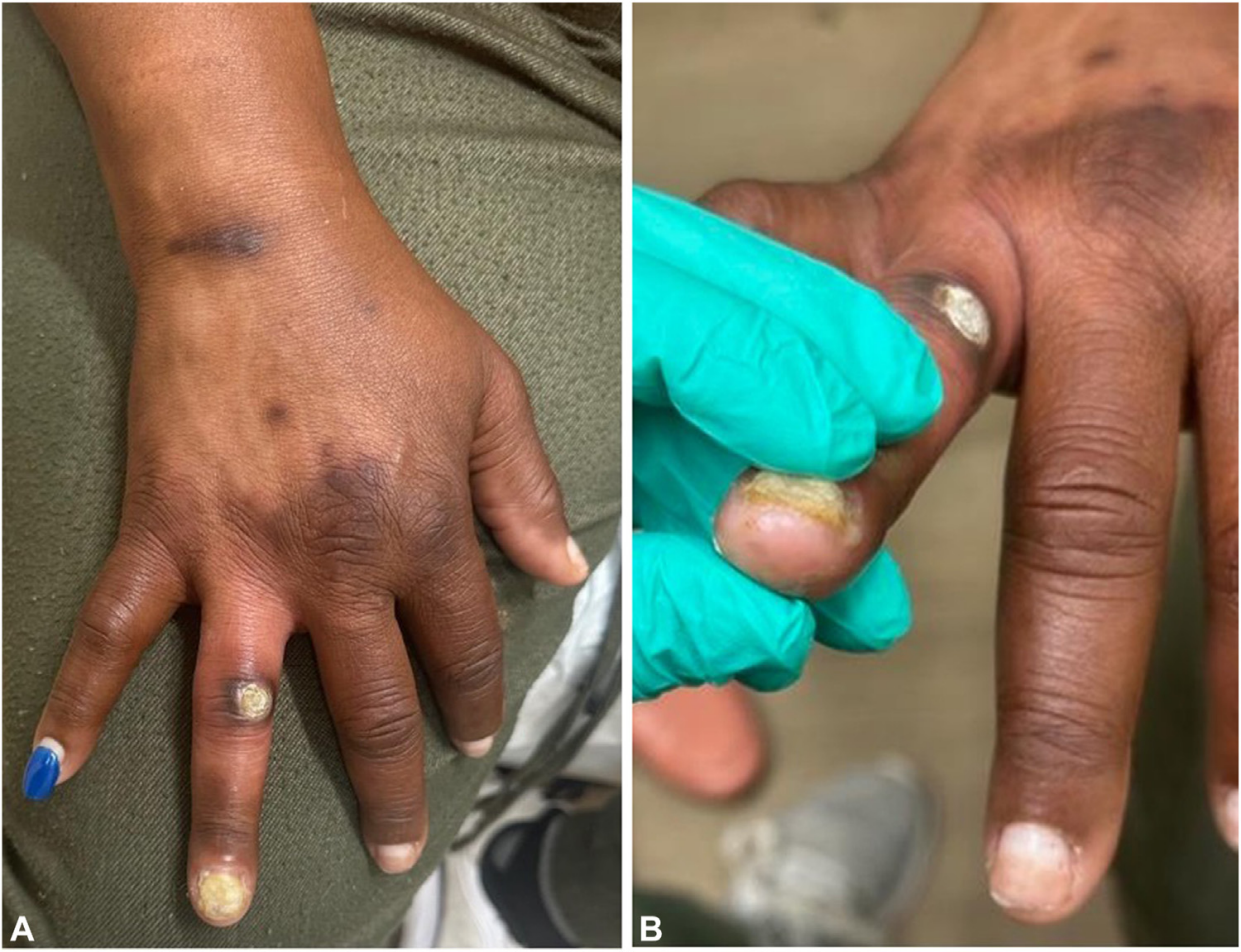
Two months after shave biopsy of the skin of the right dorsal fourth digit, the biopsy site remains unhealed (**A**), and there is dystrophy of the fourth fingernail with subungual pus (**B**). Hypopigmentation of the skin of her dorsal aspect of the hand and wrist is also noted.

Two months later, she experienced severe pain and edema of the affected digit. The skin biopsy site remained unhealed and there was subungual pus from the dystrophic fingernail (Fig 1, *A, B*), with culture growing *Pseudomonas aeruginosa* treated with ciprofloxacin.

**Fig 2:**
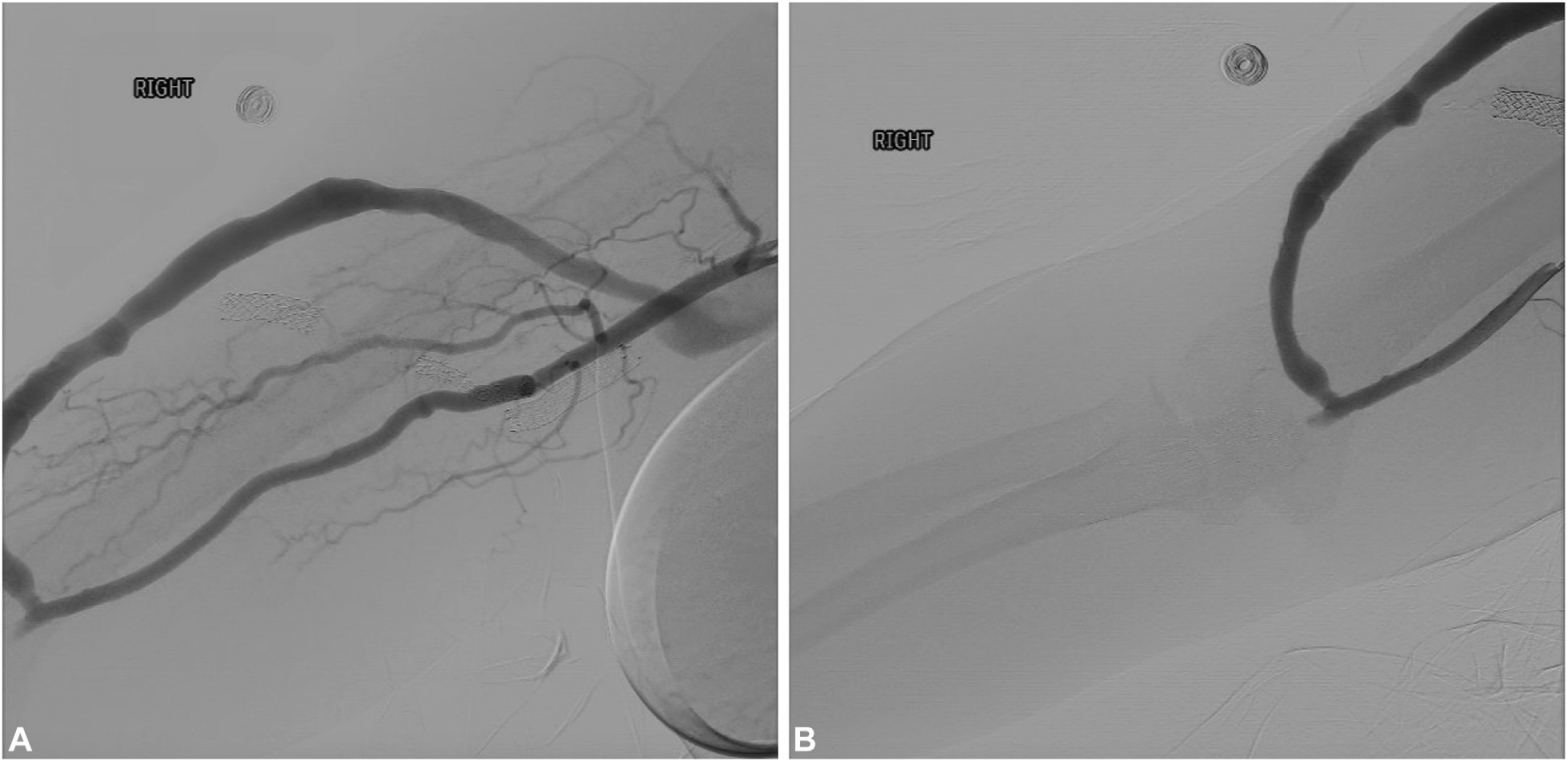
Our patient’s angiogram of the upper portion of the right arm showing rapid fill/opacification of the brachial artery and the dialysis graft (**A**). Essentially all the opacified blood is seen flowing through the dialysis graft with no blood extending into the forearm (**B**), compatible with vascular steal syndrome.

She denied trauma to the digit. The patient was referred to the hospital for pain management and further evaluation, where radiographs revealed a tuft fracture of the affected distal phalanx, suspected to be due to prolonged vascular insufficiency as severe digital ischemia is a proposed mechanism for acro-osteolysis.^3^ An angiogram of the right side of the upper extremity was performed and demonstrated reduced blood flow to the distal arm and hand (Fig 2, *A, B*), consistent with vascular steal phenomenon.

**Fig 3:**
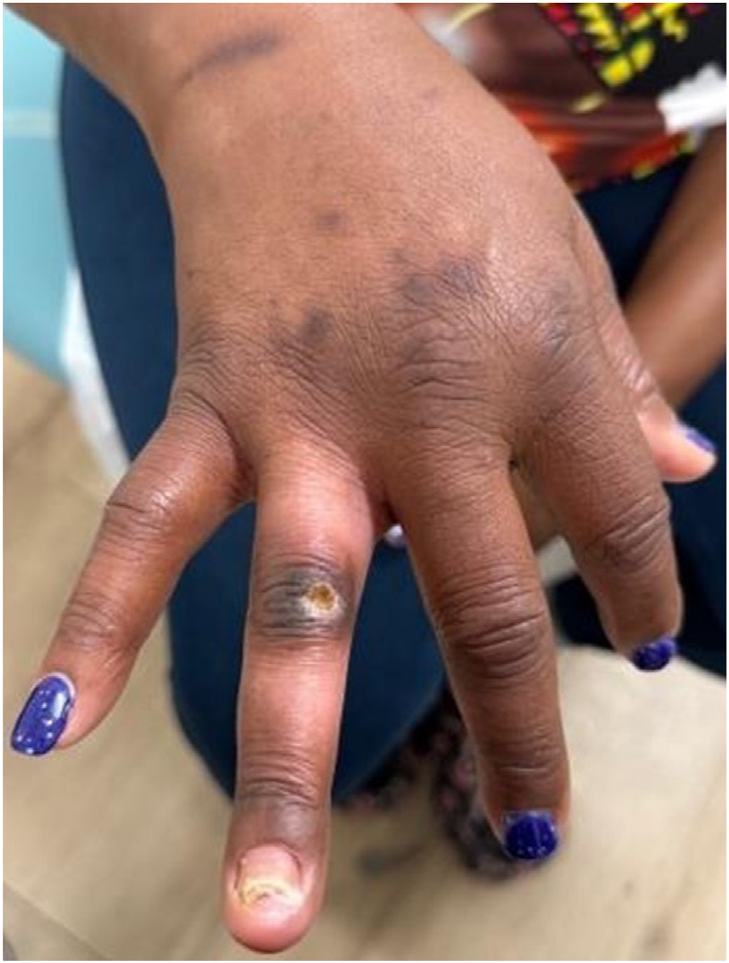
Improvement of the wound on the dorsal right fourth digit and nail dystrophy after hyperbaric oxygen therapy.

At that time, she also had significant hyperglycemia from diabetes mellitus and elevated cardiac troponins. She began daily hyperbaric oxygen therapy, leading to improved blood flow and healing of the skin lesion and fingernail but without resolution (Fig 3).

**Fig 4:**
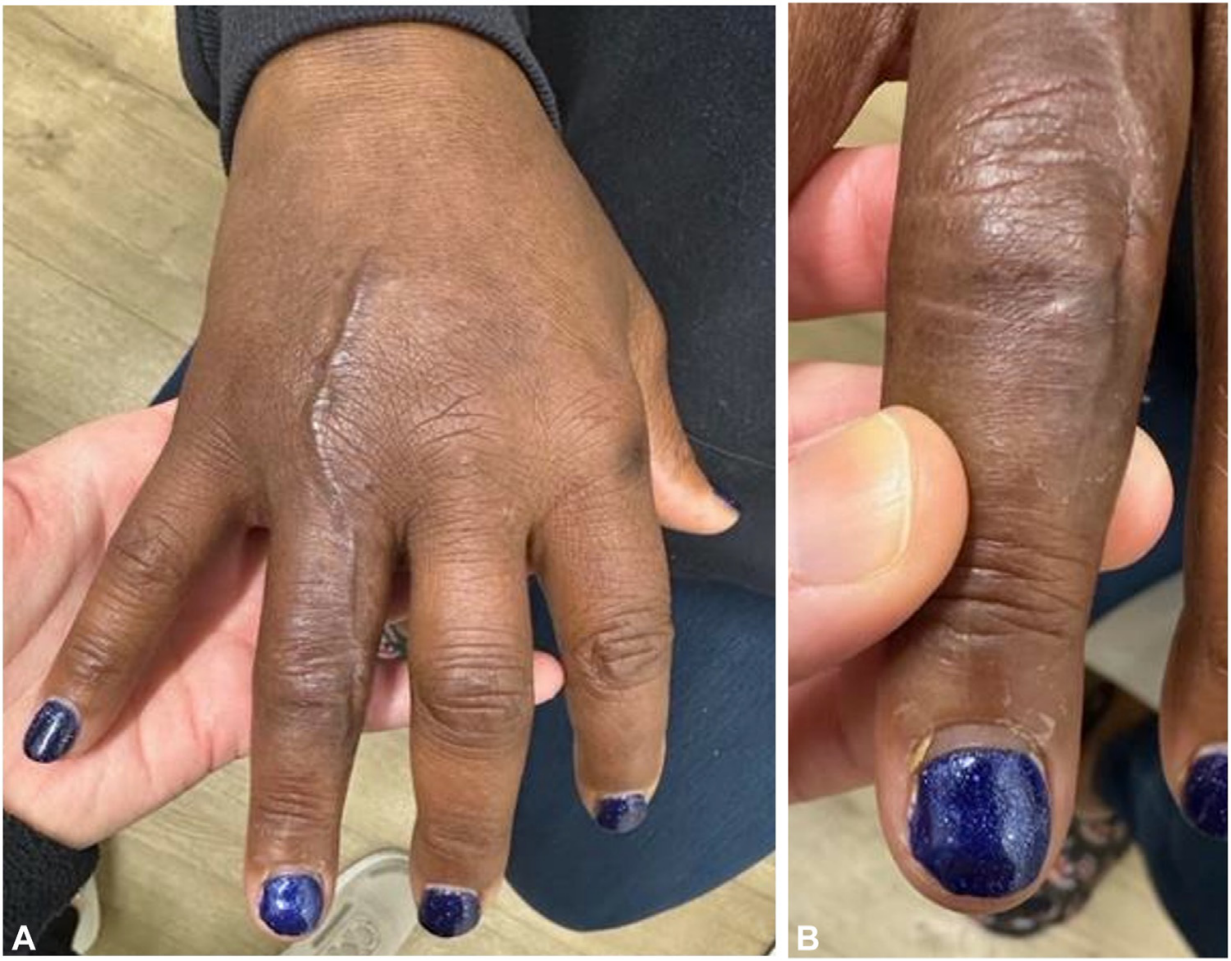
Two months after arteriovenous fistula ligation, (**A**) the skin wound was fully healed and the skin pigment returned to normal. **B,** There was normal regrowth of the fingernail noted proximally under nail polish.

The patient later underwent placental grafting of her wound, which was complicated by postoperative infection. Ultimately the AVF was ligated by vascular surgery, resulting in complete wound healing, normal regrowth of the fingernail, normal-appearing skin pigmentation, and resolution of pain at the 2-month follow-up visit (Fig 4, *A, B*). She continues physical therapy to the affected digit.

## Discussion

ESRD is associated with a multitude of cutaneous disorders, many of which are exacerbated by hemodialysis therapy.^4^ The most reported cutaneous manifestations of ESRD include pruritus, xerosis, hyperpigmentation, half and half nails, perforating disorders, calcifying disorders (including calciphylaxis), nephrogenic systemic fibrosis, and bullous dermatoses.^4^, ^5^, ^6^ DASS, a feared complication of AVF placement in patients with ESRD, may present with variable dermatologic manifestations including cyanosis, erythema, ischemic ulcers, and gangrene of the upper extremity.^5^ Onychodystrophy, which was present in our patient, has also recently been reported as a presenting sign of steal syndrome.^2^ We attributed our patient’s skin hypopigmentation of the dorsal aspect of the hand and wrist to represent postinflammatory hypopigmentation from chronic ischemia and inflammation; these pigment changes also resolved after AVF ligation. Kyrle disease (acquired perforating dermatosis) has been reported to follow a Koebner phenomenon and may have been isolated to the finger in our patient as a result of exaggerated angiopathy and oxidative stress from vascular steal syndrome.^7^

Our patient presented a challenging case with cutaneous and noncutaneous signs that collectively pointed to underlying vascular etiology from DASS. Although our patient had undergone a successful renal transplant approximately 2 years before her presentation and was no longer requiring hemodialysis, functioning asymptomatic AVFs are typically kept in place posttransplant in case of transplant failure.^8^ This maintains the potential of developing DASS.

Once DASS is identified, it is critical for the patient to undergo prompt treatment to revascularize the affected extremity and prevent progressive ischemia. Typically, the treatment of choice involves surgical ligation of the AVF to restore blood flow, however, our patient was considered high risk for surgery because of poorly-controlled diabetes and elevated cardiac troponins, therefore hyperbaric oxygen therapy was chosen as a less invasive initial treatment while her other medical conditions could be stabilized before surgical ligation of the fistula. Hyperbaric oxygen therapy involves the patient being exposed to 100% oxygen at a certain pressure (1.4 atmosphere absolute or higher) for a period of time to allow for more oxygen to enter the lungs and subsequently increase tissue perfusion.^9^ Our review of the literature yielded no other reports of hyperbaric oxygen therapy as a treatment for DASS, indicating it may have an underrecognized role in the management of high-risk patients.

Although DASS is well known in the field of vascular surgery, it is less commonly encountered by dermatologists. Clinicians must be familiar with the dermatologic manifestations of ESRD, especially in patients with AVFs. Digital pain in the setting of a nonhealing skin biopsy site and onychodystrophy should raise concern for vascular etiology. Without revascularization, many patients with DASS endure detrimental complications such as tissue necrosis and potential amputation of the affected digits.^10^ Therefore, prompt recognition of DASS by dermatologists can be potentially limb- and life-saving.

## Conflicts of interest

None disclosed.
